# Pulsed field (endocardial) ablation as part of convergent hybrid ablation for the treatment of long-standing persistent atrial fibrillation

**DOI:** 10.1007/s10840-022-01183-3

**Published:** 2022-03-19

**Authors:** Federico T. Magni, Bart A. Mulder, Michiel Rienstra, Theo J. Klinkenberg, Massimo A. Mariani, Yuri Blaauw

**Affiliations:** 1 Department of Cardiology, University of Groningen, University Medical Center Groningen, Groningen, Netherlands; 2 Department of Cardio-Thoracic Surgery, University of Groningen, University Medical Center Groningen, Groningen, Netherlands

A 69-year-old male patient with a pacemaker, previous hypertension, myocardial infarction, and long-standing persistent atrial fibrillation (AF) was referred for staged convergent hybrid ablation AF ablation. During this procedure, epicardial surgical ablation of the left atrial (LA) posterior wall (PW) is performed followed by endocardial catheter ablation of the pulmonary veins (PV) and remaining PW gaps [1]. We present a case of staged convergent hybrid ablation where pulsed field ablation (PFA) was used for the endocardial procedure.

**Fig. 1:**
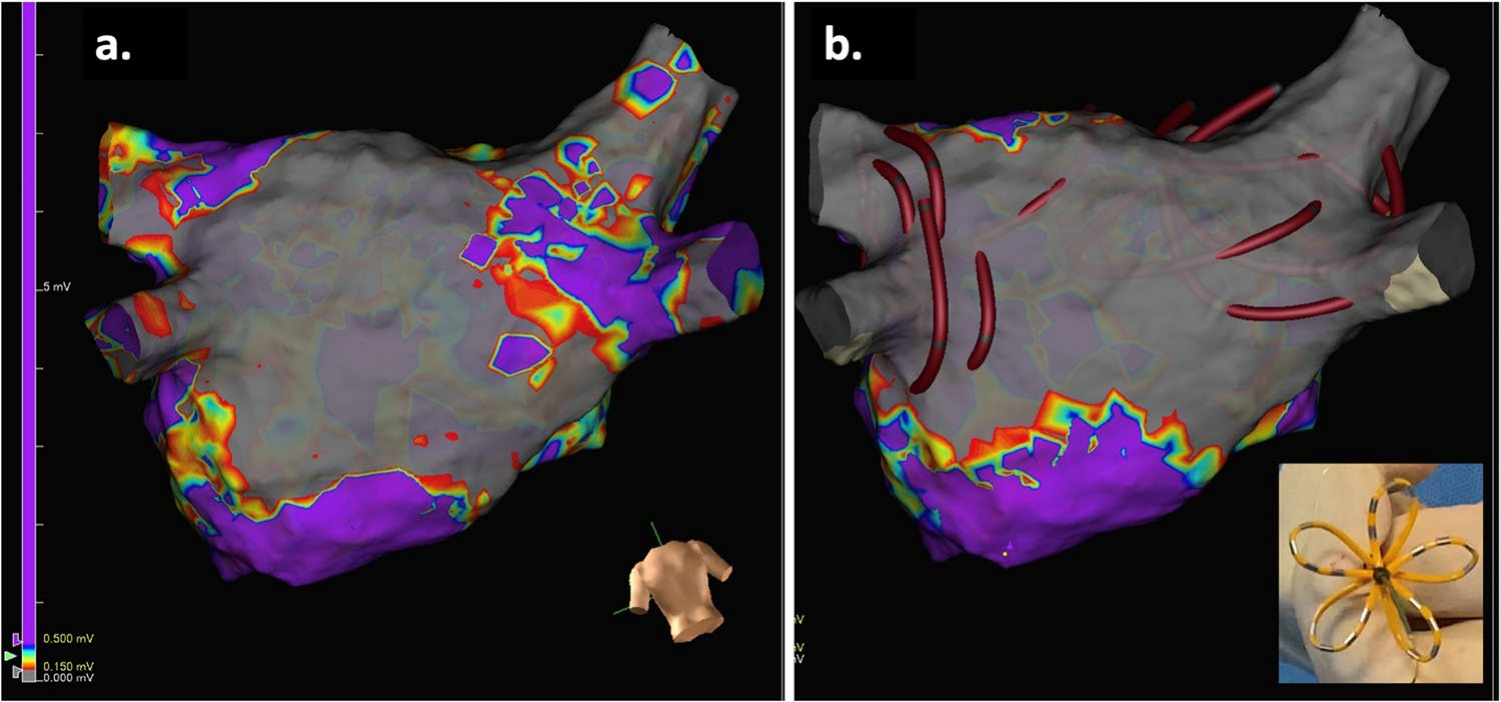
**a** Three-dimensional electroanatomic voltage map of the LA (posterior-anterior view, 0.15–0.50 mV) before PFA endocardial ablation. Low-voltage areas (highlighted in gray) represent an extensive “posterior box” lesion made during epicardial surgical ablation. This is displaced towards the inferior aspect of the posterior wall between the two inferior pulmonary veins. **b** Three-dimensional electroanatomic voltage map of the LA (posterior-anterior view) after PFA endocardial ablation. Low-voltage areas (highlighted in gray) highlight the additional pulmonary vein isolation performed and the extension of the existing posterior box to the superior aspect of the LA posterior wall. The catheter is represented in the 3D map as a circular catheter and the positions during PFA are shown. PFA applications to the LA posterior wall were delivered with the PFA catheter in the flower shape, as depicted in the right lower panel

Nine weeks after the epicardial procedure, 3D-electroanatomic mapping of the LA (ENSITE Precision) showed a large PW lesion (Fig. 1 *Panel A*). Additional PV isolation was performed with a 35-mm PFA catheter (FARAPULSE, Fig. 1 *Panel B*) with four applications in the flower and four in the basket shape for each PV. The posterior box was extended superiorly to the PW area inaccessible during surgical ablation due to pericardial reflections, with the PFA catheter in the flower position (positions are shown in *Panel B*). Remapping confirmed PV isolation and posterior box extension. No esophageal temperature probe was used. Total procedure time was 76 min.

We present the first case of convergent hybrid convergent AF ablation where PFA technology was used for endocardial ablation. PFA is a novel ablation modality with high acute efficacy, fast ablation times, and tissue selectivity, which is especially important when ablating the PW thus sparing the esophagus [2]. Here, PFA seems to offer a good alternative to radiofrequency technology.
